# Model-Based Acceleration of Look-Locker T1 Mapping

**DOI:** 10.1371/journal.pone.0122611

**Published:** 2015-04-10

**Authors:** Johannes Tran-Gia, Tobias Wech, Thorsten Bley, Herbert Köstler

**Affiliations:** 1 Department of Diagnostic and Interventional Radiology, University of Würzburg, Würzburg, Germany; 2 Comprehensive Heart Failure Center (CHFC) Würzburg, University of Würzburg, Würzburg, Germany; German Cancer Research Center, GERMANY

## Abstract

Mapping the longitudinal relaxation time *T*_1_ has widespread applications in clinical MRI as it promises a quantitative comparison of tissue properties across subjects and scanners. Due to the long scan times of conventional methods, however, the use of quantitative MRI in clinical routine is still very limited. In this work, an acceleration of Inversion-Recovery Look-Locker (IR-LL) *T*_1_ mapping is presented. A model-based algorithm is used to iteratively enforce an exponential relaxation model to a highly undersampled radially acquired IR-LL dataset obtained after the application of a single global inversion pulse. Using the proposed technique, a *T*_1_ map of a single slice with 1.6mm in-plane resolution and 4mm slice thickness can be reconstructed from data acquired in only 6s. A time-consuming segmented IR experiment was used as gold standard for *T*_1_ mapping in this work. In the subsequent validation study, the model-based reconstruction of a single-inversion IR-LL dataset exhibited a *T*_1_ difference of less than 2.6% compared to the segmented IR-LL reference in a phantom consisting of vials with *T*_1_ values between 200ms and 3000ms. In vivo, the *T*_1_ difference was smaller than 5.5% in WM and GM of seven healthy volunteers. Additionally, the *T*_1_ values are comparable to standard literature values. Despite the high acceleration, all model-based reconstructions were of a visual quality comparable to fully sampled references. Finally, the reproducibility of the *T*_1_ mapping method was demonstrated in repeated acquisitions. In conclusion, the presented approach represents a promising way for fast and accurate *T*_1_ mapping using radial IR-LL acquisitions without the need of any segmentation.

## Introduction

Quantitative evaluation of *T*_1_ relaxation times is desirable in many clinical MRI applications. One of the most prominent ways to perform *T*_1_ mapping is to track the spin-lattice relaxation process after a suitable magnetization preparation. The resulting images and a relaxation model according to the type of preparation applied are then used for a fit of the relaxation parameters. Especially for short relaxation times, which occur in many clinically relevant types of tissue, this can be difficult and measurements have to be performed in segmented fashion or with low spatial resolution [1–3]. Most conventional *T*_1_ mapping methods therefore require long acquisition times, impairing the usefulness of this promising investigation for many clinical applications.

Recently, a Model-based Acceleration of Parameter mapping (MAP) algorithm in conjunction with radial data acquisition was proposed, capable of fully resolving an exponential signal evolution after saturation recovery (SR) magnetization preparation [3]. While SR prepared Look-Locker (SR-LL) sequences can be described by a very simple two-parameter model for the relaxation process [3–5], the model only yields an effective longitudinal relaxation parameter *T*_1_^*^. Variations of the flip angle *α* within the excited slice may cause difficulties in the direct calculation of *T*_1_ [5]. By replacing the SR magnetization preparation with an inversion recovery (IR) preparation scheme, additional information about the relaxation process—namely the equilibrium magnetization *M*_0_—becomes available. While this slightly complicates the fitting procedure, the true *T*_1_ can be obtained without knowledge of the flip angle *α* [5]. To take advantage of this benefit and enable a direct quantification of *T*_1_, the MAP technique is extended to inversion recovery prepared sequences in this work. To additionally sensitize the fitted model to the influence of multi-exponential relaxation in voxels containing multiple different tissue types, a dictionary-based approach as in [6] was used instead of the more conventional mono-exponential fitting step used for the model-enforcement.

The proposed IR-MAP method allows quantifying *T*_1_ in a single slice from a radially acquired dataset collected after the application of a single IR pulse. The technique could be advantageous in many applications such as dynamic contrast-enhanced (DCE) MRI where a dynamic quantification of relaxation parameters could be used to evaluate the contrast agent uptake.

## Theory

### *T*_1_ Mapping based on the Look-Locker concept

*T*_1_ mapping sequences based on the Look-Locker (LL) concept [4] are widely used for MR parameter mapping. Typically, an IR magnetization preparation pulse is applied, followed by a series of low-angle radiofrequency (RF) pulses used for spoiled gradient echo imaging. As described in [5], the resulting longitudinal relaxation process under the influence of continuous excitation with flip angle *α* follows an effective longitudinal relaxation time *T*_1_^*^<*T*_1_ given by:

$$T_{1}^{*}={\left[1/T_{1}-\left(1/T_{R}\right)\cdot \text{ln}\left(\text{cos }\alpha \right)\right]}^{-1}$$ (1)

*T*_R_ stands for the repetition time between two consecutive excitations. Additionally, the continuous application of RF pulses leads to a steady-state magnetization *M*_0_^*^ lower than the equilibrium magnetization *M*_0_:

$$M_{0}^{*}=M_{0}\cdot T_{1}^{*}/T_{1}$$ (2)

This relation only holds for *T*_R_<*T*_1_^*^. The relaxation process in this so-called IR-LL sequence can therefore be described by:

$$M\left(t\right)=M_{0}^{*}-\left(M_{0}+M_{0}^{*}\right)\text{exp}\left(-t/T_{1}^{*}\right)$$ (3)

A specific time point *t* of the IR-LL relaxation process (i.e. after the inversion recovery magnetization preparation pulse) is typically referred to as inversion time *TI*. Consequently, a measurement of the temporal evolution of the longitudinal magnetization *M*(*t*) during the relaxation allows determining *M*_0_^*^, *M*_0_ and *T*_1_^*^ by means of a three-parameter curve fit. The desired value *T*_1_ can be calculated independently of the flip angle using a combination of Eqs. 2 and 3 [5]:

$$T_{1}=T_{1}^{*}\cdot \left[\left(M_{0}+M_{0}^{*}\right)/M_{0}^{*}-1\right]$$ (4)

### MAP Review

**Fig 1:**
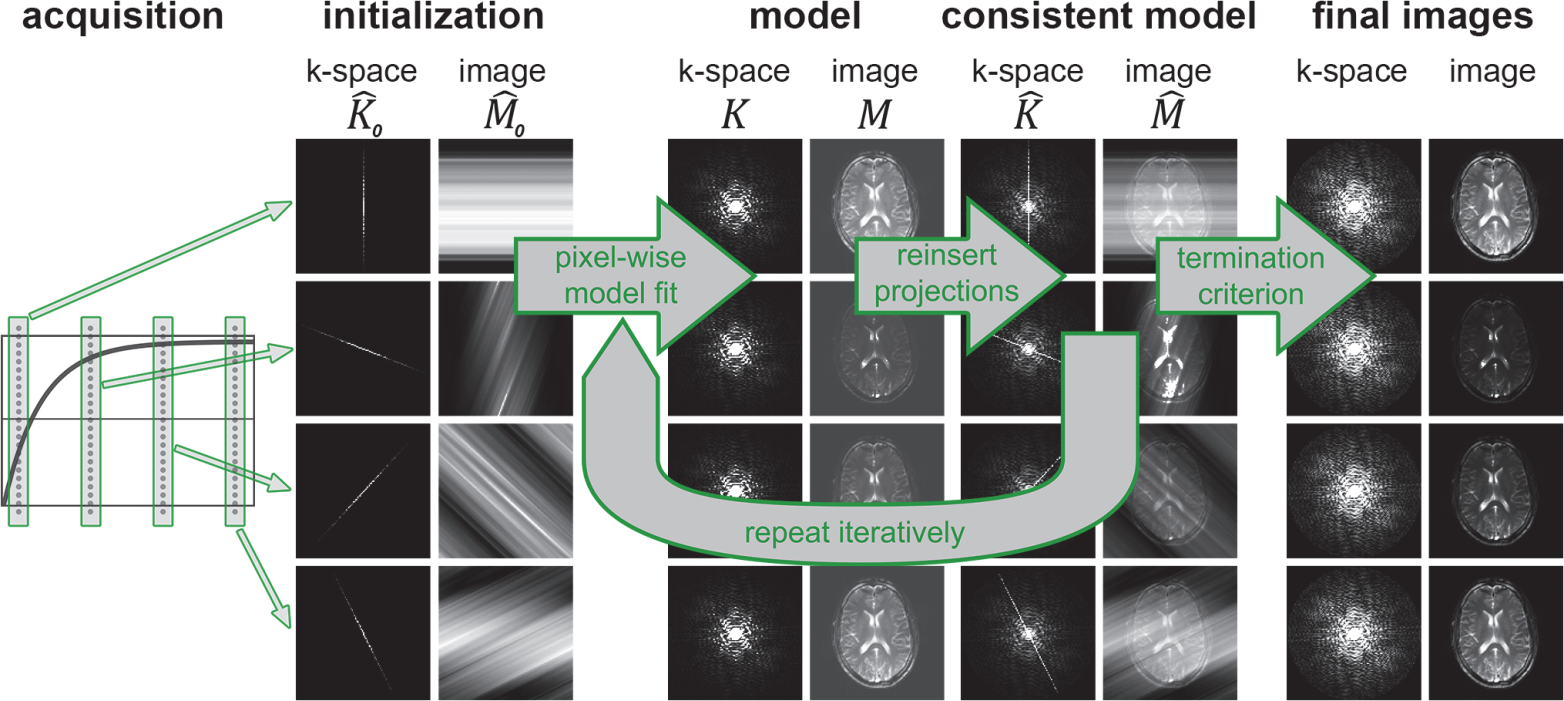
Reconstruction scheme of the algorithm. The acquisition consists of a magnetization preparation (SR for SR-MAP, IR for IR-MAP), followed by a relaxation process (solid curve) during which the projections are collected (dotted lines).

The SR prepared MAP algorithm (SR-MAP), which was introduced in [3], iteratively enforces the mono-exponential relaxation model of Eq. 3 to the data acquired in a radial SR-LL acquisition. The sequence is similar to the IR-LL sequence described above, only with a non-selective SR pulse used for magnetization preparation. The saturation pulse involves the assumption of no remaining longitudinal magnetization *M*_0_ before the subsequent radial spoiled gradient echo acquisition (i.e. *M*_0_ = 0 in Eq. 3). The resulting set of radial projections is used in an iterative reconstruction, which is schematically depicted in Fig 1. After gridding each radial projection on a separate Cartesian grid using self-calibrating GROG [7,8], one iteration consists of the following steps:

1. Inverse discrete Fourier transform of the current model k-spaces $\hat{K}\left(t\right)$ into image space $\hat{M}\left(t\right)$ (first iteration: ${\hat{K}}_{0}\left(t\right)$, ${\hat{M}}_{0}\left(t\right)$).
2. Pixel-wise two-parameter fit of Eq. 3 (*M*_0_ = 0) yielding *T*_1_^*^ and *M*_0_^*^ in every pixel. This corresponds to one 2D model image *M*(*t*) for every time point *t*.
3. Discrete Fourier transform to generate the corresponding model k-spaces *K*(*t*).
4. Ensure data consistency: Substitute original for the model data for all acquired projections. This results in the consistent model k-spaces $\hat{K}\left(t\right)$ that are passed on to the next iteration.

The result is one *T*_1_^*^ as well as one *M*_0_^*^ value for each pixel which can be depicted in a spatially resolved map describing the local relaxation behavior. A more detailed explanation of the SR-MAP algorithm can be found in [3].

### IR-MAP for a direct determination of *T*_1_

A considerable drawback of the SR-MAP method (used in conjunction with an SR preparation) is that it can only be used to determine the effective relaxation parameters *T*_1_^*^ and *M*_0_^*^. As stated above, a direct computation of *T*_1_ from *T*_1_^*^ using Eq. 1 is difficult because of variations of the flip angle *α* across the excited FoV. To enable a direct determination of *T*_1_ using a similar model-based reconstruction, the magnetization was prepared with a global inversion. In consequence, the three-parameter model of Eq. 3 has to be applied, yielding *T*_1_^*^, *M*_0_^*^ and *M*_0_ in every pixel. After termination of the adjusted algorithm, which will be called IR-MAP in the following, these three parameters can be used with Eq. 4 to obtain the desired *T*_1_ map independently of the flip angle.

### Improvement of the Initial Estimates

In the reconstruction scheme in Fig 1, solely the gridded k-spaces of the single projections are used as initial estimates ${\hat{K}}_{0}\left(t\right)$. To improve the convergence behavior of these “nearly empty” k-spaces, improved estimates were generated by performing a linear interpolation of all acquired k-space points through time. Using these “filled” k-space as initial model ${\hat{K}}_{0}\left(t\right)$ greatly improves the time-efficiency of IR-MAP.

### Sensitizing IR-MAP to multi-exponential relaxation

According to [3,6,9,10], the quality of iterative model-based reconstruction techniques highly depends on the accuracy with which the acquired signal is described by the model. Even small systematic deviations between the model and the measured data can accumulate to considerable errors in the reconstruction or even prevent convergence of iterative techniques. In model-based relaxometry, potential sources of error include multi-exponential relaxation in voxels containing multiple different tissue types (i.e. multiple *T*_1_ values) as well as inaccuracies in the excitation profile, resulting in a flip angle distribution rather than a single flip angle in each voxel [9].

In order to sensitize the fitted model to these influences, a dictionary-based approach similar to [6] was used instead of a mono-exponential fit of Eq. 3 for the model-enforcement in step 2 of the algorithm. First, a dictionary is created consisting of a set of mono-exponential relaxation curves calculated using Eq. 3 for 740 different parameter combinations of *T*_1_ (185 values between 10ms and 5000ms) and *α* (3°, 5°, 7°, 9° for a nominal flip angle of *α* = 7°). Next, an orthogonal matching pursuit (OMP, [11]) algorithm is used in conjunction with this dictionary to find a linear combination of signal prototypes best describing the relaxation behavior in each voxel of the consistent model images $\hat{M}\left(t\right)$. This dictionary-based multi-exponential model fit serves for improving the accordance between the measured data and the fitted model and thereby minimizing model violations as they are described above. With this modification of the fitting step 2, multiple iterations of the algorithm are executed.

After termination, the result is an image series that is based on a linear combination of several relaxation curves of different parameter sets (*T*_1_, α). As described above, the dictionary-based modeling in the presented approach is only used for the purpose of describing the relaxation curve as exactly as possible. To make the presented approach comparable to conventional IR-LL *T*_1_ mapping, a regular mono-exponential fit of Eq. 3 is applied to the final series of consistent model images in a pixel-wise fashion. Using Eq. 4, one single *T*_1_ value is calculated for every pixel out of the resulting parameters *T*_1_^*^, *M*_0_^*^ and *M*_0_.

### Using the IR-MAP algorithm with multiple receiver coils

For data acquisition with multiple receiver coils, the sensitivity profiles *c*_*γ*_(*j*) of the signal in pixel *j* received in coil *γ* at time point *t* were included in the signal model in Eq. 3, leading to:

$$M_{\gamma }\left(j,t\right)=c_{\gamma }\left(j\right)\cdot M\left(j,t\right)$$ (5)

In [3], these single coil signals were combined using a sign-independent sum of squares (SoS) approach to obtain coil-combined consistent model images $\hat{M}\left(j,t\right)$ for the fitting step 2 of the algorithm. In contrast to an SR relaxation process, where only positive values are to be expected, an inversion can lead to positive and negative values. The current complex-valued consistent model images ${\hat{M}}_{\gamma }\left(j,t\right)$ of all coils (which are described by the model *M*_*γ*_(*j*,*t*) in Eq. 5) were therefore combined using a sign-dependent SoS approach which was implemented as follows: To obtain real-valued curves, a phase map *φ*_*γ*_(*j*) for each pixel *j* and each coil *γ* was calculated from a fully sampled image obtained from the last 200 projections of the LL-IR measurement—the Nyquist limit for a radial acquisition with 128 readout points per projection. At this point of the relaxation, the magnetization has already passed the zero-crossing and is assumed to have reached the steady-state magnetization *M*_0_^*^ with a constant contrast behavior. With the resulting phase map *φ*_*γ*_(*j*), a real-valued inversion recovery relaxation curve for each coil γ was obtained by taking the real part of the complex-valued magnetization ${\hat{M}}_{\gamma }\left(j,t\right)$ rotated to the real axis:

$${\hat{M}}_{\text{Re},\gamma }\left(j,t\right)=\text{Real}\left\{{\hat{M}}_{\gamma }\left(j,t\right)\cdot \text{exp}\left(-i\varphi_{\gamma }\left(j\right)\right)\right\}$$ (6)

To combine these relaxation curves for all coils, a sign-dependent SoS was calculated using

$${\hat{M}}_{\text{SoS}}\left(j,t\right)=\text{sign}\left(\theta \left(j,t\right)\right)\cdot \sqrt{\left|\theta \left(j,t\right)\right|}$$ (7)

with

$$\theta \left(j,t\right)=\sum_{\gamma }\left[\text{sign}\left({\hat{M}}_{\text{Re},\gamma }\left(j,t\right)\right)\cdot {\left|{\hat{M}}_{\text{Re},\gamma }\left(j,t\right)\right|}^{2}\right]$$ (8)

These real-valued IR-LL relaxation curves were used for the dictionary-based model fit in step 2 of the algorithm, resulting in the signal model *M*(*j*,*t*).

In order to substitute the original data for data consistency (step 4 of the algorithm), this model had to be re-separated into single coil relaxation curves *M*_*γ*_(*j*,*t*). The complex factor *c*_*γ*_(*j*) in Eq. 5 describes how the fitted signal model *M*(*j*,*t*) splits into real and imaginary part of a single coil model image *M*_*γ*_(*j*,*t*). Therefore, *c*_*γ*_(*j*) was calculated separately for each coil *γ* using a least squares approach of Eq. 5 on the current consistent model images ${\hat{M}}_{\gamma }\left(j,t\right)$. The result was a set of complex-valued model images *M*_*γ*_(*j*,*t*) which was Fourier transformed into k-space (step 3), where the data consistency (step 4) was performed to obtain new consistent model k-spaces ${\hat{K}}_{\gamma }\left(j,t\right)$ for the next iteration.

## Methods

### Numerical implementation & Hardware

The algorithm was implemented in MATLAB (The MathWorks, Natick, MA). All reconstructions were performed on an Intel Core i7-2600 CPU (3.4 GHz). For comparability of the reconstructions, the number of iterations was set to a fixed number of 50 for all IR-MAP reconstructions performed in this work.

All imaging experiments were carried out on a 3T whole-body scanner (MAGNETOM Trio, Siemens AG Healthcare Sector, Erlangen, Germany) employing a 12 channel phased-array head coil for signal reception. Data and reconstruction code (in MATLAB) are available under http://dx.doi.org/doi:10.5061/dryad.165r8.

### Phantom measurements

**Fig 2:**
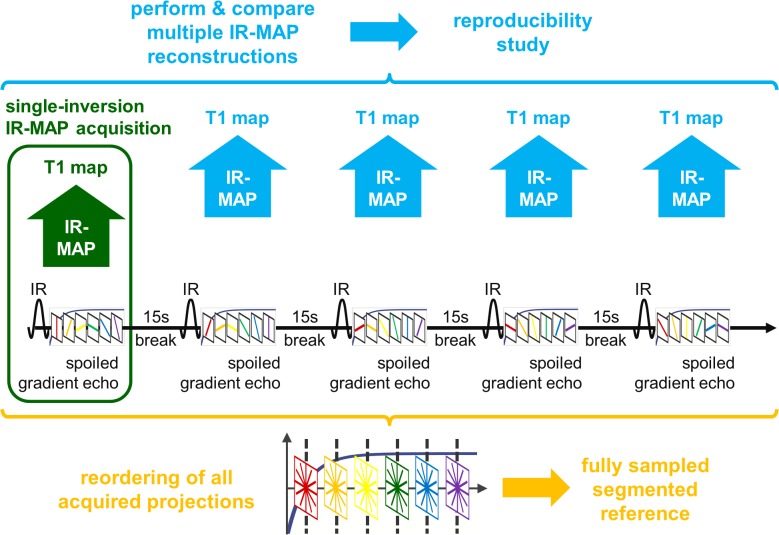
Acquisition scheme of all acquisitions performed in this work. The regular IR-MAP acquisition consists of one inversion followed by a spoiled gradient-echo data collection (green). The segmented reference is acquired by performing multiple IR-LL acquisitions, each of which is followed by a 15s break for relaxation before the next inversion (orange). Using IR-MAP reconstructions for multiple of the acquired IR-LL datasets, a reproducibility study was carried out (blue).

In order to validate the functionality of the proposed reconstruction algorithm and the accuracy of the resulting *T*_1_ values, a phantom study was performed on a phantom consisting of seven vials with different concentrations of contrast agent (Resovist, Bayer Schering Pharma, Berlin, Germany) and copper sulfate (CuSO_4_). *T*_1_ measurements were performed with a globally prepared IR-LL sequence (FoV = 200×200mm^2^, slice thickness: 4mm, *T*_E_ = 2.5ms, *T*_R_ = 6ms, *α* = 7°, total acquisition time: 6s) with a Golden Ratio [12] radial k-space trajectory (1000 projections, 128 readout points per projection). After the acquisition, 50 iterations of the IR-MAP algorithm were applied for the model-based reconstruction, with the dictionary fit limited to a linear combination of three mono-exponential relaxation signals. After obtaining *T*_1_^*^, *M*_0_^*^ and *M*_0_ in the final mono-exponential fitting step, Eq. 4 was used to compute a *T*_1_ map out of these parameters. This single-inversion IR-MAP acquisition is schematically depicted in Fig 2 (green).

To assess the accuracy of these relaxation times, a fully sampled IR-LL reference was acquired in a segmented fashion which is schematically depicted in Fig 2 (orange). The acquisition consisted of 200 consecutive IR-LL measurements—the Nyquist limit for a radial measurement with 128 readout points per projection. While the same IR-LL sequence as before was used, the order of the 1000 projections was varied for each inversion pulse. After reordering these projections, the result was a set of 1000 k-spaces, each carrying the contrast of one of the 1000 inversion times *T*_I_ used in the acquisition and each fully sampled with 200 projections. Each of these IR-LL segments was followed by a delay of 15s, allowing for a complete relaxation before the acquisition of the subsequent IR-LL segment. This led to a total scan time of ~1h compared to 6s for the single inversion measurement. After gridding, a pixel-wise three-parameter fit of Eq. 3 to the SoS of all coils was used to obtain reference *T*_1_^*^, *M*_0_^*^, *M*_0_ maps. Again, Eq. 4 was used to obtain *T*_1_.

Using the data of the segmented reference acquisition, a reproducibility study was performed by separately applying IR-MAP reconstructions to the first ten IR-LL segments. This is illustrated in Fig 2 (blue). The resulting set of *T*_1_ maps was used to test the reproducibility of the *T*_1_ values obtained from the IR-MAP reconstruction in repeated acquisitions.

Additionally, an inversion recovery *T*_1_ mapping experiment with only one RF excitation and radial gradient-echo readout per inversion was performed (FoV = 200×200mm^2^, matrix size: 128×128, slice thickness: 4mm, *T*_E_ = 2.5ms, *T*_R_ = 6ms, *α* = 7°, total acquisition time: ~10h). In a segmented fashion, fully sampled images were acquired for ten inversion times (13ms, 50ms, 100ms, 250ms, 500ms, 1000ms, 1600ms, 2500ms, 5000ms, 8000ms). As in the segmented IR-LL measurement, a relaxation break of 15s was kept between successive segments. After the acquisition, a fit of Eq. 3 resulted in a *T*_1_ map that was used for validating the general accuracy of the IR-LL technique.

A ROI analysis was used to obtain mean values μ_vial_ and standard deviations σ_vial_ of *T*_1_ in every compartment of both the reference and the IR-MAP reconstructed *T*_1_ maps. Additionally, the signal-to-noise ratio (SNR) in each of the vials was calculated using SNR_vial_ = *μ*_vial_/*σ*_vial_ to compare the SNR of all three reconstruction techniques [13].

### In vivo measurements

The study was approved by our local ethics committee (Ethics Committee at the Faculty of Medicine, University of Würzburg, reference no 22/11). Written informed consent was obtained from each volunteer prior to scanning.

*T*_1_ measurements of the brains of seven healthy volunteers aged between 23 and 30 years were carried out with the same sequence as in the phantom experiments (slice thickness: 4mm, *T*_E_ = 2.5ms, *T*_R_ = 6ms, *α* = 7°, 1000 projections, 128 readout points per projection, total acquisition time: 6s), with a FoV ranging between 200×200mm^2^ and 220×220mm^2^. After the acquisition, *T*_1_ maps were obtained using IR-MAP as described in the phantom experiments.

Additionally, a fully sampled IR-LL reference dataset was obtained using the same segmented acquisition as in the phantom experiments. To shorten the scan time, the number of segments was reduced from 200 to 100, leading to each of the 1000 contrast images consisting of only 100 projections, the equivalent of a two-fold Nyquist under-sampling. Maintaining a relaxation delay of 15s between successive inversions, the total scan time was reduced to ~30min. Using this acquisition, a reference *T*_1_ map was obtained as described in the phantom experiments.

A ROI analysis was used to obtain mean values of *T*_1_ in white matter (WM), grey matter (GM) and cerebrospinal fluid (CSF) for the reference as well as the IR-MAP reconstructed *T*_1_ maps of each volunteer. For comparison of both reconstruction methods, a mean SNR over all volunteers was obtained as described in the phantom experiments.

## Results

### Phantom measurements

**Fig 3:**
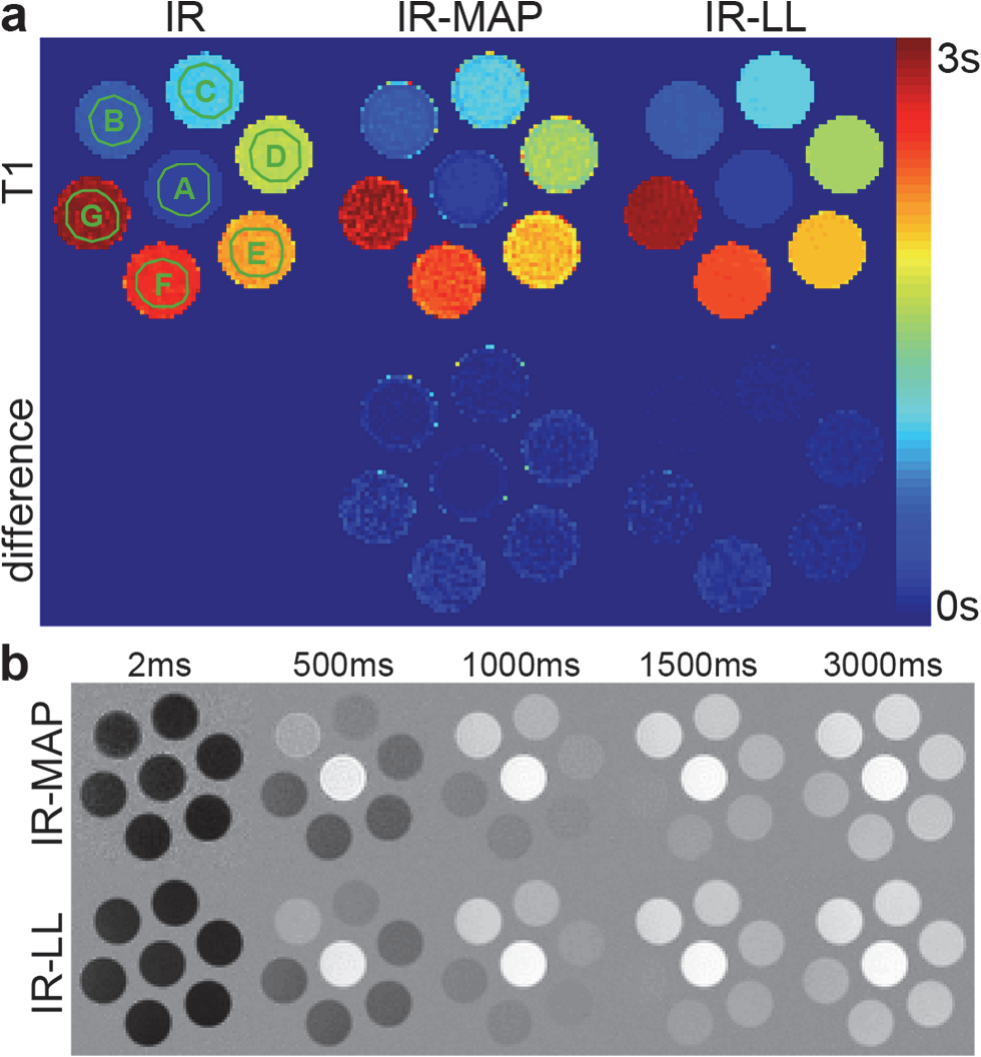
Phantom measurement. a: T_1_ maps of the segmented IR reconstruction (left), the IR-MAP reconstruction of the single-inversion IR-LL acquisition (center) and the segmented IR-LL reference (right) and the differences (bottom). b: Reference IR-LL images and IR-MAP reconstructions for exemplary inversion times.

Fig 3 shows the results of the phantom measurement. The top row of Fig 3A depicts the masked *T*_1_ maps obtained from the segmented IR reference (left), the IR-MAP reconstruction of a single-inversion IR-LL measurement (center) as well as the segmented IR-LL reference (right). The bottom row shows differences of both IR-LL-based reconstructions from the segmented IR reference. A comparison of the two reference measurements shows a small deviation between the segmented IR-LL and the segmented IR method increasing with *T*_1_. Despite a noisier appearance, the same deviation is visible for the IR-MAP reconstructed *T*_1_ map. The visual observations are confirmed by the associated ROI analysis depicted in Table 1. The noisier appearance of the IR-MAP reconstruction is confirmed by a lower SNR (ranging between 15 and 35) especially in comparison to the segmented IR-LL acquisition (ranging between 80 and 220), but also to the segmented IR acquisition (ranging between 31 and 72). However, despite the larger standard deviations of the IR-MAP reconstruction, the values of both IR-LL-based methods are in good agreement with a difference smaller than 2.6% for all ROIs. Although there is a larger deviation between both of these methods and the segmented IR measurement, the differences, which are additionally listed in Table 1, also stay below 5.2% for all ROIs. Overall, the IR-MAP results are in very good agreement with both references.

**Table 1:** ROI analysis of the phantom study.

**Vial**		**A**	**B**	**C**	**D**	**E**	**F**	**G**
**IR**	**T1 (ms)**	208±4	573±15	998±20	1659±23	2123±38	2560±49	2929±94
	**SNR**	50	38	49	72	55	52	31
**IR-MAP**	**T1 (ms)**	202±13	574±35	1048±37	1579±55	2064±59	2427±70	2894±129
	**SNR**	15	16	29	28	35	34	22
	**diff (%)**	2.7	0.2	5.0	4.8	2.8	5.2	1.2
**IR-LL**	**T1 (ms)**	197±2	560±6	1046±6	1574±8	2042±9	2418±13	2885±22
	**SNR**	80	101	171	188	220	191	130
	**diff (%)**	5.2	2.3	4.8	5.1	3.8	5.5	1.5

Listed are means and standard deviations of T_1_ within the ROIs A-G of the phantom experiment as well as the corresponding SNR. For the two IR-LL-based acquisitions, the percentage difference to the IR values is additionally listed.

Fig 3B shows images of the IR-MAP reconstruction (top) and the reference (bottom) for exemplary inversion times. Again, although the visual appearance of the IR-MAP reconstructions is noisier, the contrasts of the both reconstructions are in good agreement.

**Fig 4:**
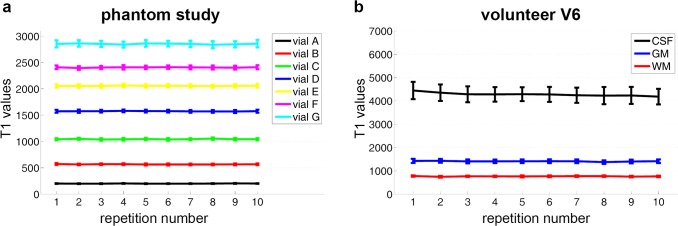
Reproducibility study. Shown are the mean T_1_ values obtained from IR-MAP reconstructions of ten consecutive IR-LL acquisitions, each of which was followed by a 15 second break for relaxation. While a) shows the results in the seven ROIs of the phantom (A-G), b) depicts the ROIs used for evaluation of T_1_ in WM, GM and CSF of volunteer V7.

The reproducibility study, which is depicted in Fig 4A, shows no systematic deviations in means and standard deviations of *T*_1_ in repeated IR-MAP reconstructions of the same FoV.

### In vivo measurements

**Fig 5:**
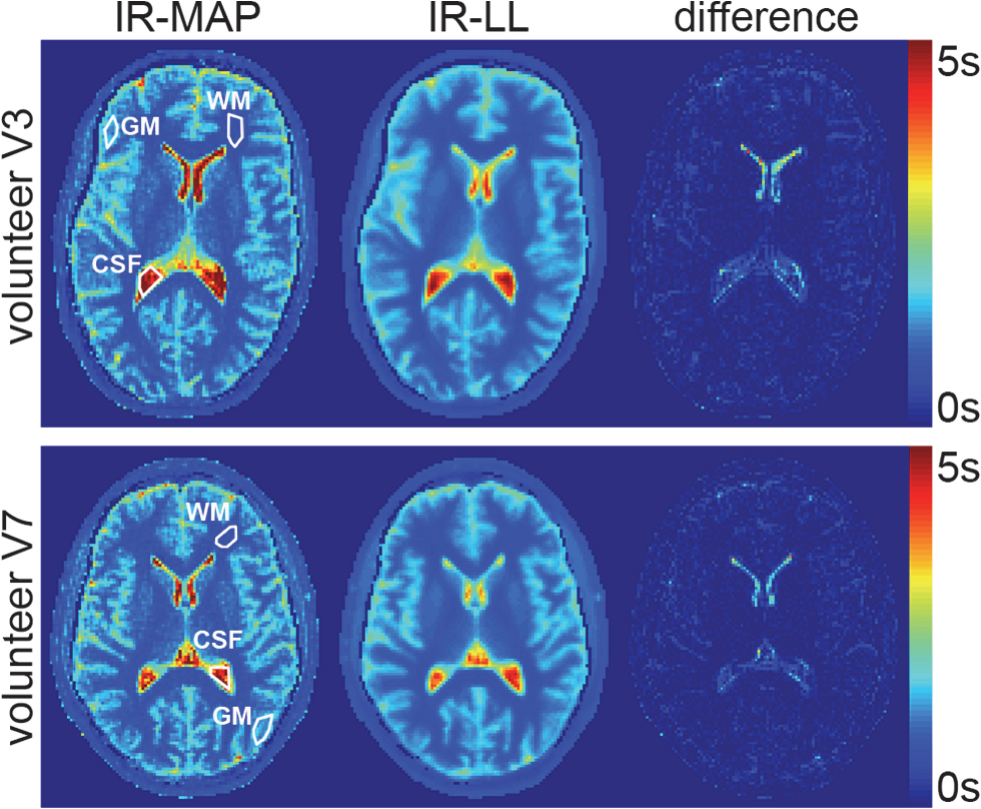
T_1_ maps of the IR-MAP reconstruction (left) and the segmented IR-LL reference (center) as well as a difference (right) for volunteers V3 and V7.

**Fig 6:**
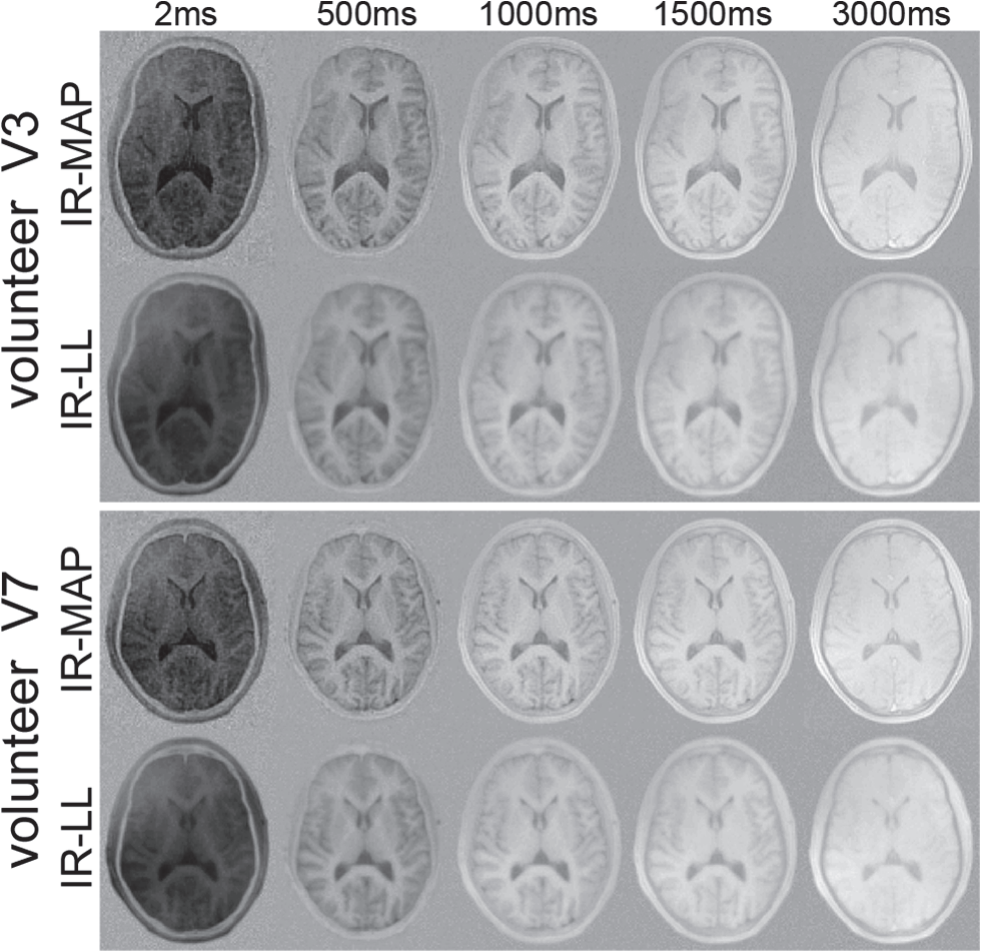
In vivo images. IR-MAP reconstruction and the segmented IR-LL reference of volunteers V3 and V7 at exemplary inversion times.

The results of the in vivo measurements are depicted in Figs 5 and 6. Fig 5 shows *T*_1_ maps from IR-MAP reconstructions (left) and the IR-LL reference (center) as well as a difference for volunteers V3 and V7. Despite a general accordance between both *T*_1_ maps, the IR-MAP *T*_1_ maps have the same noisier overall appearance that was already observed in the phantom measurements. In contrast to the phantom study, blurring can be observed in the in vivo reference. The largest differences occur in small areas of large *T*_1_ values such as the CSF in the ventricles.

Fig 6 shows the corresponding images of the IR-MAP reconstruction (top) and the reference (bottom) for the same exemplary inversion times as in the phantom experiments. Despite the blurry nature of the reference images that was already observed in the *T*_1_ maps, both reconstructions reveal the same contrast variations. The visual impression is confirmed by the results of the ROI analysis in Table 2. The noisier appearance of the IR-MAP reconstruction manifests in a larger standard deviation within the ROIs as well as a smaller mean SNR. This difference decreases with the value of *T*_1_ (SNR_CSF_ < SNR_GM_ < SNR_WM_). However, differences of the means in WM and GM stay below 5.5% in all volunteers, underlining the good agreement between the two reconstructions. In contrast, the means in the CSF regions deviate by up to 25.3% in V6.

**Table 2:** Means and standard deviations obtained in the ROI analysis of the in vivo measurement (in ms).

	**White Matter (WM)**		**Grey Matter (GM)**		**Cerebrospinal fluid (CSF)**	
	**IR-MAP**	**REF**	**IR-MAP**	**REF**	**IR-MAP**	**REF**
**V1**	738±82	734±16	1447±127	1436±94	4603±657	4296±494
**V2**	706±76	709±42	1405±85	1385±98	4586±670	4082±620
**V3**	740±68	712±21	1458±159	1402±158	4473±720	3877±700
**V4**	688±78	695±31	1453±207	1378±147	3991±508	3924±348
**V5**	679±82	693±17	1429±286	1401±192	4387±925	4061±911
**V6**	726±99	698±17	1400±90	1400±58	4055±361	3236±249
**V7**	779±63	744±11	1430±178	1409±71	4359±726	3878±564
**mean**	722±78	712±22	1432±162	1402±117	4350±651	3908±555
**SNR**	9	32	9	12	7	7

The ROIs were placed in white matter (WM), grey matter (GM) and cerebrospinal fluid (CSF) areas of the various T_1_ maps of all seven volunteers (V1-V7). The different T_1_ values correspond to the IR-MAP reconstruction and the fully sampled reference (REF). The last two rows depict the means and standard deviations over all volunteers as well as the corresponding SNR.

The in vivo reproducibility study, which is depicted in Fig 4B, shows no systematic deviations in means and standard deviations of *T*_1_ in WM and GM. However, *T*_1_ in the CSF is decreasing with each of the repeated IR-MAP acquisitions.

## Discussion

### Phantom measurements

The *T*_1_ maps of the phantom experiments clearly prove the functionality of IR-MAP for parameter mapping. The lower SNR of the IR-MAP reconstructions can be explained by the fact that only a small part of the data measured for both references were used in the acquisition (0.5% for the IR-LL reference and 10% for the IR reference). This SNR reduction also causes the higher standard deviations within the ROIs of the IR-MAP reconstructed *T*_1_ maps. With a difference of less than 2.6% to the IR-LL reference and 5.2% to the IR reference, IR-MAP still provides a very good accuracy of the reconstructed *T*_1_ values. The images obtained by the IR-MAP reconstruction reveal the same noise enhancement as the *T*_1_ maps. Apart from that, the images feature the same spatial and temporal resolution as well as the same contrast as the reference. Altogether, the very small degradation in image quality and the slightly larger standard deviation of the determined *T*_1_ values is acceptable considering the acquisition time of 6s for the IR-MAP reconstruction compared to ~1h for the IR-LL reference and ~10h for the IR reference.

The reproducibility study demonstrates the functionality of the IR-MAP method in repeated measurements.

### In vivo measurements

All observations regarding the noise-increase of the IR-MAP reconstructions compared to the reference can be explained as in the phantom measurements. Due to the nature of radial sampling patterns, the two-fold under-sampling of the reference dataset should only lead to negligible streaking artifacts and only a small loss in spatial resolution. Therefore, it was assumed that the additionally appearing blurriness of the reference *T*_1_ maps resulted from motion of the volunteers, which is unavoidable for the lengthy acquisition of ~30min. This hypothesis is confirmed by the considerably sharper appearance of the IR-MAP *T*_1_ maps, each of which were acquired within only 6s and which are therefore much less susceptible to motion artifacts. If regions of high *T*_1_ values extend to only one or two pixels, motion of the subject smears out each of these values over a small region of several pixels, leading to lower *T*_1_ values in these regions. Due to the very fast acquisition process, less motion can occur in IR-MAP reconstructions, leading to much less pronounced blurring and therefore an improved accuracy in *T*_1_.

The reproducibility study showed no changes in WM and GM for repeated measurements of the same FoV. The decreasing *T*_1_ values in the CSF can be explained by the relaxation breaks of only 15s that were used to ensure a complete relaxation of the magnetization before every IR-LL measurements. While 99.9% of the magnetization will be relaxed for *T*_1_<2000ms, less than 98% will be relaxed for *T*_1_>4000ms, leading to errors in a quantification of *T*_1_ using Eq. 4. In conjunction with the motion-induced *T*_1_ reduction mentioned above, this effect explains the smaller *T*_1_ values observed in the CSF of the in vivo reference. With a proper relaxation time, the reproducibility of IR-MAP could be ensured even in regions of higher *T*_1_ such as the CSF. Such an acquisition, however, would be impractical for in vivo studies due to unacceptably long acquisition times.

Although there is a wide range of literature GM and WM values, the *T*_1_ values of GM obtained in this study (ranging from 1395ms to 1455ms) are in good agreement to many literature values [14–16] with a range from 1331ms to 1470ms. Although the values of WM (ranging from 676ms to 777ms) are smaller than many literature values for overall WM such as 832ms in [17], it is in agreement with the value of 761ms found in [16] for frontal white WM. Due to the large standard deviation within the ROIs of the CSF, these values were not compared to other literature.

### Comparison of IR-MAP and SR-MAP

The proposed IR-MAP algorithm addresses some of the major issues of the initially presented SR-MAP algorithm. The most important change is the IR pulse which is used instead of the SR pulse for magnetization preparation. Instead of an effective relaxation parameter *T*_1_^*^, one can now obtain *T*_1_ without an explicit determination of the flip angle. Additionally, the use of IR pulses results in an SNR gain in comparison to SR prepared sequences, where the magnetization is initially zeroed. This can lead to a very low SNR especially for the radial projections acquired directly after the SR pulse and therefore causes an inevitable introduction of noise by the data consistency condition in step 4 of the algorithm. A drawback of the IR preparation is the additional relaxation time needed for reaching the equilibrium magnetization before subsequent IR-LL acquisitions which was already addressed in the previous section. This leads to a considerably longer scan time if multiple subsequent parameter maps are to be acquired.

Another limitation of the initial SR-MAP implementation which is not connected to the type of magnetization preparation used was the assumption of a mono-exponential relaxation in every voxel. Despite the advantage of a low numerical complexity of the fit, this can lead to systematic errors in image regions or voxels not complying with this model such as voxels containing various types of tissue [3]. To avoid such problems, a multi-exponential fit of up to three mono-exponentials was allowed in the dictionary look-up used in the IR-MAP implementation. In addition, imperfect slice profiles caused by the very short excitation pulses typically used in fast imaging sequences can cause variations of the flip angle *α* within the excited slice [18]. This problem was addressed by adding mono-exponentials of different flip angles to the dictionary used for the model fit.

### Dependence of the accuracy of the *T*_1_ estimates on the temporal coverage

As described for the initial MAP algorithm [3], the exponential slope as well as the steady-state magnetization *M*_0_^*^ have to be covered in every pixel to ensure a quantification of *T*_1_ with a sufficient accuracy. All sequence parameters have to be chosen accordingly to fulfill this condition. For a repetition time *T*_R_ = 6ms and a flip angle *α* = 7°, which was used in the experiments of this work, *M*_0_^*^ would be covered even for very large *T*_1_ values of about 4000ms as they would only occur in tissues with an extremely high water content such as the CSF. This ensures an accurate quantification of *T*_1_.

### Duration of the IR-MAP algorithm

In our implementation, one iteration of the dictionary-based algorithm lasted 90s for a dataset with 1000 time steps *T*_I_ and a 256×256 image matrix. In comparison, a mono-exponential fit of the same matrix would take 86s on the same CPU using standard MATLAB libraries. In both cases, this time is proportional to the number of pixels in the image matrix. For dictionary-based model fitting, the reconstruction time additionally depends on the size of the dictionary. A straightforward and therefore quite lengthy implementation of the dictionary look-up was used in this work to demonstrate the functionality of the method. However, the duration of the look-up for an entire dataset can be reduced by starting with a broad spacing of the parameters *T*_1_ and *α* used in the dictionary of the first look-up which is recursively reduced in multiple iterations. As the look-up is performed in a sequential fashion for the individual pixels, there is also a great potential of speeding up the algorithm by parallelizing the implementation.

### Comparison of IR-MAP to existing *T*_1_ mapping techniques

Many other *T*_1_ mapping techniques have been proposed, some of which achieve full brain coverage with 1mm in-plane resolution in less than 10mins. Most techniques based on the LL approach apply inversion pulses, followed by a LL acquisition, to track the LL relaxation process and subsequently use a fit similar to Eq. 4 to obtain a *T*_1_ map. For a sufficiently high temporal and spatial resolution, the acquisition is performed in a segmented fashion using multiple inversions [19–21]. These approaches offer whole brain coverage with a slice thickness of up to 2mm and an in-plane resolution of 1mm with a total acquisition time of less than 10mins, leading to an effective acquisition time of about 20s per slice. However, due to the segmented acquisition of the entire volume, the data needed to obtain the *T*_1_ map of a single slice are collected after multiple sequential inversion pulses. Therefore, the temporal resolution for obtaining the *T*_1_ map of a single slice using these techniques would therefore be considerably worse than the 6s achieved using IR-MAP.

Another popular way of *T*_1_ mapping enabling an even better spatial resolution are techniques based on the variable flip angle (VFA) approach [22]. Using this approach, the 3D acquisition of a whole brain *T*_1_ map with an isotropic resolution of 1mm is possible in less than 8mins [23]. These measurements require the exact knowledge of the flip angle, leading to potential errors due to B_1_ field inhomogeneities and slice profile imperfections. Due to the broad excitation profiles of fast RF pulses typically used in 2D acquisitions, a reliable *T*_1_ map with a slice thickness of 1mm would be hard to obtain using a VFA technique. As the slice profile errors are relatively low in the center portion of 3D slabs, most implementations of the VFA technique use 3D acquisitions. Similar to the LL based approaches, the temporal resolution of the *T*_1_ map of a single slice therefore corresponds to the acquisition time of the entire 3D volume. Moreover, the performance of VFA methods that use spoiled gradient echo acquisitions is dependent on the value of the RF increment used for RF spoiling [24], which has to be corrected for [25].

In contrast to the methods described above, IR-MAP has a slightly lower spatial resolution of up to 1.6mm × 1.6mm × 4mm, but no segmentation is necessary, enabling a self-contained acquisition of a single-slice *T*_1_ map in 6s. This could be advantageous for applications such as dynamic *T*_1_ mapping where a better temporal resolution is required.

## Conclusion

A model-based acceleration of parameter mapping using Inversion-Recovery prepared Look-Locker (IR-LL) sequences is introduced. The presented IR-MAP technique enables the reconstruction of a *T*_1_ map of a single slice with 1.6mm in-plane resolution and 4mm slice thickness from data acquired in only 6s.

The IR-LL-based *T*_1_ mapping approach used in this work was successfully validated by a comparison to a segmented inversion recovery *T*_1_ mapping experiment with only one RF excitation and gradient-echo readout per inversion—the gold standard in *T*_1_ mapping using relaxometry. A subsequent comparison of the model-based IR-MAP reconstructions of single-inversion IR-LL datasets to segmented IR-LL reconstructions demonstrated the functionality of the presented IR-MAP technique in the phantom and in vivo. Except for the inevitable noise enhancement caused by the extremely high acceleration, all IR-MAP reconstructions were of comparable visual quality as the fully sampled IR-LL references. A ROI analysis resulted in *T*_1_ differences smaller than 2.6% in the phantom and 5.5% in WM and GM of seven healthy volunteers. Finally, the repeatability of the presented method was proven both in the phantom and in vivo.

All in all, IR-MAP represents a promising way for extremely fast *T*_1_ mapping from radial IR-LL datasets without the need of any segmentation.
